# Thin Copper Foils: From Electrodeposition Conditions to Adhesion Performances

**DOI:** 10.3390/ma19091838

**Published:** 2026-04-29

**Authors:** Ivana O. Mladenović, Željko Radovanović, Dana G. Vasiljević-Radović, Rastko Vasilić, Miloš Vorkapić, Predrag Živković, Nebojša D. Nikolić

**Affiliations:** 1 Institute of Chemistry, Technology and Metallurgy, University of Belgrade, Njegoševa 12, 11000 Belgrade, Serbia; dana.vasiljevic@ihtm.bg.ac.rs (D.G.V.-R.);; 2 Innovation Center of Faculty of Technology and Metallurgy Ltd., 11000 Belgrade, Serbia; zradovanovic@tmf.bg.ac.rs; 3 Faculty of Physics, University of Belgrade, Studentski Trg 12-16, 11000 Belgrade, Serbia; rastko.vasilic@ff.bg.ac.rs; 4 Faculty of Technology and Metallurgy, University of Belgrade, Karnegijeva 4, 11000 Belgrade, Serbia

**Keywords:** electrodeposition, copper, molybdenum, stainless-steel, additive technology, adhesion

## Abstract

**Highlights:**

- The copper films were produced by the electrodeposition in galvanostatic regime on Mo and 316L SS substrate.
- The electrodeposition of copper was performed from solutions without and with added suppressor/activator additives.
- The adhesion of Cu films from the cathodes was estimated by a lab-made bending test machine made by additive technology.
- The worst adhesion, i.e., the easiest delamination, was observed for nanocrystalline mirror-bright films.
- Both Mo and 316L SS cathodes fulfilled the conditions of easy delamination of the films from them.

**Abstract:**

Cathodic electrodeposition of copper on molybdenum and stainless-steel substrates has been investigated with the aim of examining their potential to produce thin copper foils (TCFs). Copper in the form of a thin film was electrodeposited galvanostatically from the acidic sulfate electrolyte without and with an addition of suppressor/activator additives, such as chloride ions, polyethylene glycol 6000 and 3–mercapto–1–propanesulfonic acid. The cathodes and electrodeposited Cu films were characterized by SEM, AFM, and XRD techniques, while the adhesion of Cu films, as a crucial parameter in the production of Cu foils, was estimated by a lab-made prototype of a bending test machine made by applying additive technology. The adhesion parameter named “critical cycle number” (*n*_c_), which defines the minimal number of cycles leading to a delamination (separation) of the film from the cathode was used for assessing the adhesion features of the films. The easiest delamination, i.e., the smallest *n*_c_, showed nanocrystalline films obtained with the addition of all additives, whereupon the values were significantly smaller than the values obtained for microcrystalline films obtained without and with a partial combination of the additives. The easy delamination of the nanocrystalline films indicated that both substrates have a high potential for application in the production of TCFs.

## 1. Introduction

Thin copper foils (TCFs) can be synthesized in different ways whereupon each of them has certain limitations. The main processes are rolling (with a limitation on a thickness range and mechanical stress), chemical vapor deposition–CVD (very expensive process and utilization of specialized equipment), and physical vapor deposition–PVD (it has slow deposition rate) [1]. TCFs are an important material applied in modern energy storage devices, particularly in lithium-ion batteries [2], collectors [3], and supercapacitors [4]. Among various fabrication methods, electroplating has emerged as the most effective route for producing TCFs with controlled microstructure and tailored properties. Recent advances focus on the production of ultra-thin Cu foils with a smooth surface, improved conductivity, and enhanced adhesion to active materials, enabling a higher energy density and a longer cycle life [5,6,7]. One of the ways to obtain a foil with a high tensile strength and a low roughness is through the use of a combination of functionalized ionic liquids without/with additives [8] and sulfur-based additives [9].

Thanks to high energy density, long life cycle, and reliability, Li-ion batteries have become the dominant energy storage systems for use in electric vehicles and portable electronic devices [10,11]. In Li-ion batteries, Cu foils serve as a current collector, and they play a pivotal role in ensuring efficient electron transport and are responsible for the mechanical stability of the electrode [9]. As Li-ion batteries continue to evolve toward higher energy density, improved cycling stability, and enhanced safety, the requirements imposed on copper foils have grown increasingly stringent [12]. Cu foils need to exhibit enhanced resistance to fracture under high pressure, a property that is essential for preserving the structural integrity of batteries and alleviating the adverse impact of stress concentration [13]. In particular, the trend toward reducing foils’ thickness (electroplated or rolled produced) demands simultaneous improvements in conductivity, tensile strength, elongation, and surface roughness [14]. These parameters are critical not only for maintaining electrical performance but also for ensuring strong adhesion between the current collector and active materials, thereby preventing delamination and mechanical failure during repeated charge–discharge cycles.

The actual research in a production of metal-based current collectors (M-CCs) by electroplating are partly focused on optimizing the electrodeposition conditions [15], such as solution composition [16], addition of the different additives in a solution [17], temperature, cathode choice, and selection of the seed layer [18], with the aim of obtaining metal coatings/films of superior mechanical properties [19,20,21,22,23]. The advancements are essential for meeting the challenges posed by next-generation Li-ion batteries, especially those employing high-capacity anode materials such as silicon, which undergo significant volume changes during cycling. Consequently, the development of high-performance electrodeposited copper foils is of crucial importance, since it enables us to obtain durable, safe, and high-energy-density energy storage devices.

The additives to a sulfate solution are critical for achieving defect-free, bottom-up filling of trenches and vias in the damascene process [24]. The copper electroplating in “damascene processes” has been a key technology in semiconductor manufacturing for over a decade, enabling void-free filling of high aspect ratio (HAR) trenches [24]. This is achieved with carefully designed solutions containing suppressor and accelerator additives that control the deposition rates at the via opening and bottom. Common baths include conventional sulfate, high-throughput, via-filling, and advanced additive chemistries. Typical additives in copper–sulfuric acid solutions are the chloride ions (Cl^−^), polyethylene glycol (PEG, suppressor), and 3-mercapto-1-propanesulfonic acid (MPSA, accelerator), which are often used together to a balance the electrodeposition kinetics [24]. In addition to a solution composition, the current regime and mixing conditions strongly influence the properties of electrodeposited Cu film [25,26], i.e., on grain refinement [21], hardness [20,21,22,23,24,25,26,27], electrical resistivity [27], adhesion [20], and wettability properties [22].

Conventional studies of copper electroplating are primarily focused on achieving adherent coatings with high mechanical integrity and good adhesion at the substrate–film interface [20,22,27]. In contrast, electrochemical synthesis of Cu in a form of foil demands a weak adhesion with the cathode surface, pointing out the choice of the cathode material as being very relevant to the production of current collectors. One of the ways for easy separation of TCF from the cathode is the use of an intermediate layer, but the problems with partial delamination or contamination of foil are possible in this case [28]. Wei et al. [28] prepared Cu foil (very thick ≈ 1.1 μm) using chitosan (with a hydroxyl group as the organic stripping layer). Yang et al. [29] achieved ultra TCF using discrete Cr nano-size nuclei as a release layer. The introduction of release sub-layers, although effective, alters the Cu electrodeposition process and complicates the subsequent separation of the foil. Moreover, the role of electrolyte additives in governing the structural and morphological characteristics of TCF has not been systematically investigated, leaving a critical gap in the current understanding. Finally, the majority of reported studies have been conducted under static solution conditions and at relatively low current densities, which differ substantially from industrial production environments and limit the direct applicability of the findings. Also, the production of TCFs or nano-twinned Cu (nt-Cu) by the formation of an interlayer demands various techniques for synthesizing the intermediate layer, such as magnetic sputtering. This method is expensive, reduces the thickness of the subsequent Cu layer, and results in randomly oriented grains and increased potential for migration [30,31].

Investigations into the production of two-phase copper foils were also carried out. Copper/graphene TCFs were synthesized using reverse pulse electrodeposition [32] in order to obtain extremely hard metal matrix foils that have high elasticity and retain their electrical properties. However, the problem with the strengthening second phase is that it changes the chemical composition of the TCFs.

TCFs electrodeposited on the Ti cathode often delaminate easily due to weak interfacial bonding, hydrogen evolution during deposition, and the smooth surface of Ti, although Ti is a widely used material [33]. In contrast to conventional planar TCFs, the porous variant with a hierarchical pore structure can be obtained on graphite and used to manufacture porous current collectors [34].

Using the well-established fact that the adhesion of electroplated Cu films strongly depends on the kind of cathodic material used, the main goal of this work was to synthesize Cu foils using Mo and stainless-steel, as novel types of the cathode materials applied in the production of Cu foils. Mo was chosen owing to chemical stability and conductivity, while 316L SS represents an industrial-relevant material with excellent corrosion resistance and mechanical strength. The use of these substrates is based on the expected weak adhesion of electrolytically produced films on them, by which they would fulfill conditions that can be used as cathodes in the production of Cu foils. Also, they belong to different types of crystal lattice (Mo belongs to a body-centered cubic (BCC) lattice, while 316L SS belongs to a face-centered cubic (FCC) lattice), and one of the aims would be to analyze the effect of the type of crystal lattice on the adhesion of Cu films, and hence, on the formation of Cu foils. This approach does not use sub-layers or other reinforcing phases exploiting the nature of the two incoherent materials and the crystallographic differences between the cathode and the film to easily delaminate TCFs. Cu films were electrodeposited from a basic acidic sulfate solution and with added functional additives (Cl^−^, PEG 6000, MPSA) to the sulfate solution. To test the separation of Cu films from the cathode, a laboratory prototype machine based on the principle of cyclic bending was developed for the rapid assessment of the adhesion characteristics of the films. The Cu foils were characterized using scanning electron microscopy (SEM), atomic force microscopy (AFM), and X-ray diffraction (XRD) techniques to elucidate the morphological, topographic, structural, and textural features of synthesized TCFs. The influence of additive chemistry on wettability and its implications on a foil performance under realistic environmental exposure will be discussed, aiming to identify optimal electrochemical parameters for high-quality TCFs production.

## 2. Materials and Methods

### 2.1. Chemicals and Materials

The stainless-steel 316L sheet, 430 (AMS 5627), denoted as 316L SS, was purchased from (K&S Precision Metals, Chicago, IL, USA), and Mo sheet, >99.9 wt. % purity, was purchased from Stanford Advanced Materials (SAM), Santa Ana, CA, USA, and they were used as cathodes in this study. Copper foil (99.99%), 1 mm thick, was purchased from Thermo Fisher GmbH, Kandel, Germany, and it is used as anode. An amount of 96% *v*/*v* of sulfuric acid (H_2_SO_4_) was purchased from CARLO ERBA Reagents, Milan, Italy. The copper-(II)-sulfate pentahydrate (CuSO_4_ × 5 H_2_O), sodium chloride (NaCl) > 99.0%, 3-Mercapto-1-propanesulfonic acid sodium salt, MPSA, (HSCH_2_CH_2_CH_2_SO_3_Na), and polyethylene glycol MW 6000 (PEG) were purchased from Sigma Aldrich Co. (St. Louis, MO, USA). All chemicals for the preparation of the electroplating solutions were used without additional purification.

### 2.2. Electrodeposition Conditions and Electrolyte Recipes

**Scheme 1:**
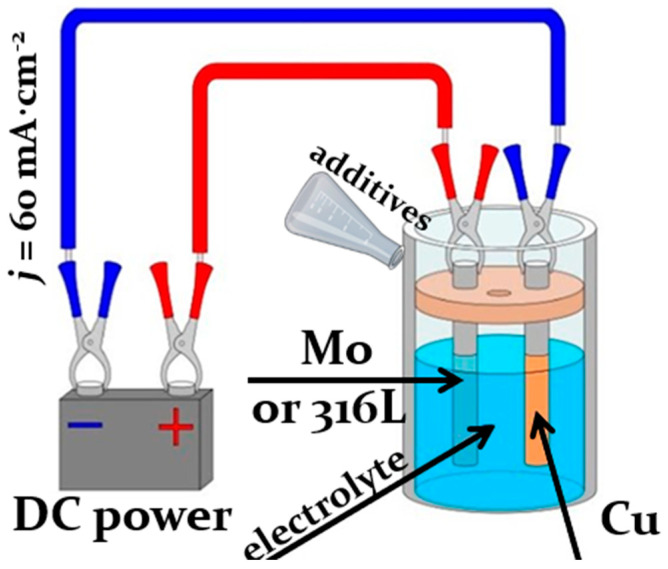
The schematic diagram of the electrodeposition process.

The electrochemical deposition (ED) of Cu was carried out in a galvanostatic mode on the Mo and 316L SS cathodes. The Mo or 316L SS cathodes surface area of (1 × 1) or (1 × 11) cm^2^ (for bending test) were put between two parallel anodes of Cu in a cylindrical cell. All parameters of the electrodeposition, including compositions of the solutions, are summarized in Table 1. The method for the preparation of the electroplating solutions and Mo and 316L SS cathodes is given in Supplementary Material S2.2. The schematic diagram of the electrodeposition process is shown in Scheme 1.

The chemical composition of selected commercial cathodes (Mo and 316L SS) is shown in Table S1, see S2.2. Additional information about the materials and methods is in the Supplementary Material.

### 2.3. Characterization Methods

Copper films were characterized by the following techniques: the surface morphology (field emission scanning electron microscope (FE-SEM)), topography and roughness analysis (atomic force microscope (AFM)) and structure (X-ray diffraction (XRD)).

The sessile drop method [22,23] and Yang–Laplace pressure for Cu films was calculated according to the procedure given in reference [35]. Additional information about the use of these techniques is given in the Supplementary Material in Section S2.3.

An automated “cyclic bend test machine”, constructed by additive technology in lab-made conditions, was used to evaluate the adhesion of Cu films. The working principle of this device is based on the critical number of cycles, *n*_c_, leading to a separation (delamination) film from the cathode. It is based on bending a sample, i.e., film around a mandrel of defined diameter, and it is described in Section 2.4. The machine’s components were printed on a 3D printer.

### 2.4. Application of Additive Technology for Construction Lab-Made “Cyclic Bending Test Machine”

A lab-made device was fabricated using a WANHAO Duplicator i3Plus (Wanhao Precision Casting Co., Ltd., Jinhua, China) 3D printer (build volume 200 × 200 × 180 mm, layer resolution 0.1–0.4 mm). Printing was performed with poly-lactic (PLA) filament (3D Republika, Belgrade, Serbia) at a plate temperature of 60 °C and nozzle temperature of 215 °C, with 100% infill density and orientations of 90/−45/0/+45 [36]. The first- and second-layer thicknesses were 0.30 mm and 0.10 mm, respectively, with a wall thickness of 1.2 mm.

**Figure 1:**
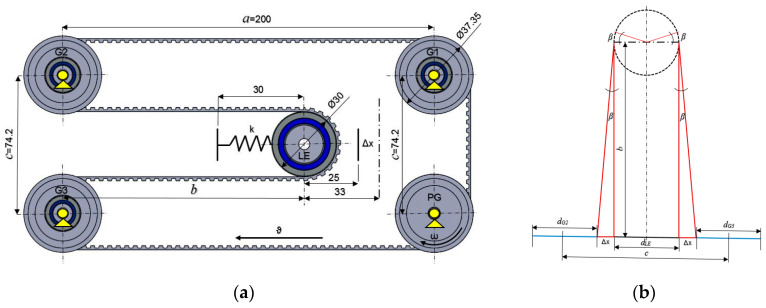
Cyclic bending test machine: (**a**) the moving mechanism, and (**b**) characteristic dimensions.

The scheme of the moving mechanism of the constructed test machine is given in Figure 1a, with characteristic dimensions for each element (Figure 1b).

**Figure 2:**
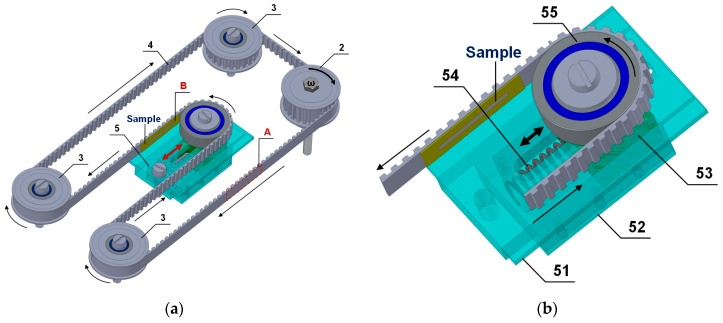
Cyclic bending test machine: (**a**) sample trajectory during bending test, and (**b**) timing belt mechanism for tensioning and adjustment.

The “cyclic bending test machine” was constructed with a toothed timing belt transmission consisting of four pulleys, a belt, and a moving mechanism. The optimal belt length was 900 mm, using a trapezoidal T5 profile reinforced with steel wires for flexibility and a resistance to elongation. One drive pulley (LS27 T5/24-2 Hub Ø26 × 6, MISUMI Europa GmbH, Frankfurt, Germany) was employed. The moving mechanism incorporates a spring-loaded tensioner with a bearing, enabling controlled belt tension, a reduced shaft and bearing load, and smooth sample bending without crushing the samples. Samples were fixed to the belt surface with double-sided adhesive tape and transported between positions A and B (Figure 2), undergoing repeated bending cycles until film detachment from a substrate occurred. The stepper motor drives the power transmission pulley (2), which turns the belt over the auxiliary pulleys (3) and the tensioner (5). The sample is placed in position A, as seen in Figure 2a. Due to the stepper motor action, the sample travels from position A to position B, thus completing one measurement cycle. The cycle includes bending the sample, first from its bottom side and then from its top side. When the sample passes over the tensioner pulley, it is ensured that there is no material kneading. The moving mechanism that supports the tensioner pulley consists of the enclosure (51, 35, 53), the spring (54), and the sliding element with a bearing (55), which enables unhindered sample movement, see Figure 2b. When the sample hits the tensioner pulley, the movable mechanism compresses the spring with the thickness of the sample. The sample with the foils applied bends and follows the outer shape of the bearing to the exit. The cycles are repeated until the foils separate or split from the flexible substrates. In this process, the moving mechanism enables a lower load for the bearing and shaft, the controlled tension of the timing belt, quieter operation, and the possibility for quick mounting of the unstrained belt on the pulleys.

**Figure 3:**
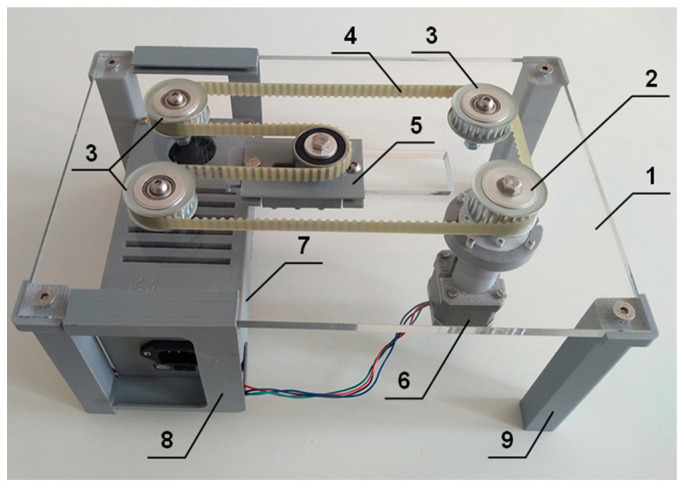
The real automatic cyclic bending test machine prepared for a delamination of Cu foils with elements: 1—work plate, 2—timing, belt pulley (power), 3—timing belt pulley (follow), 4—timing belt, 5—tensioner pulley, 6—drive assembly with stepper motor, 7—power supply control unit, 8—holder for power supply control unit and 9—legs.

A bipolar stepper motor NEMA 17HS4401 (MS MOTOR, Nanjing, China) is used as a power unit with a 1.80 angular pitch, holding torque of 0.4 N·m, and 1.7 A of the phase current (Figure 3, element 6). The stepper motor provides precise positioning of the timing belts (elements 3 and 4) and is computer controlled via a microcontroller board (element 7) connected to a stepper motor driver. The position of the stepper motor and real lab-made constructed device for the delamination of TCF was given in Figure 3. All gray elements shown in Figure 3 are designed and manufactured in a laboratory at the Institute of Chemistry, Technology and Metallurgy, Belgrade, Serbia.

## 3. Results

### 3.1. Characterization of Mo and 316L SS Substrates

**Figure 4:**
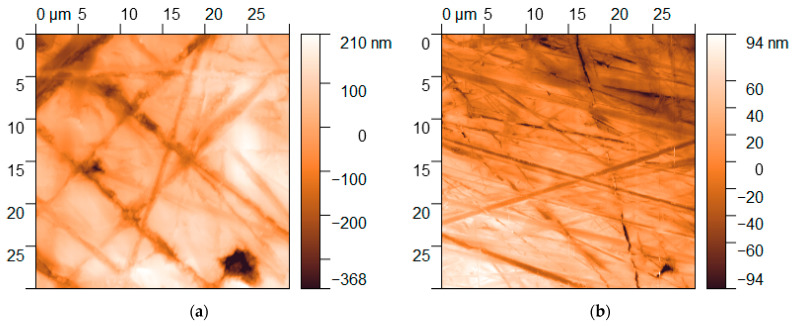
The two-dimensional (2D) AFM images of cathodes: (**a**) Mo and (**b**) 316L SS recorded after mechanical sanding. Scan size: (30 × 30) μm^2^.

Figure 4 shows 2D AFM images of Mo (Figure 4a) and 316L SS (Figure 4b) substrates used as cathodes in ED processes of Cu for obtaining TCFs. The *S*_a_ roughness (the arithmetic average of the absolute roughness) parameters of the cathodes were 78.0 nm for Mo and 25.6 nm for 316L SS.

The quantitative surface examination indicated that the Mo substrate exhibited a significantly larger *S*_a_ roughness parameter than the 316L SS substrate. This difference signifies that Mo has a more uneven topography, with prominent peaks and valleys, while 316L SS has a much smoother surface profile.

**Figure 5:**
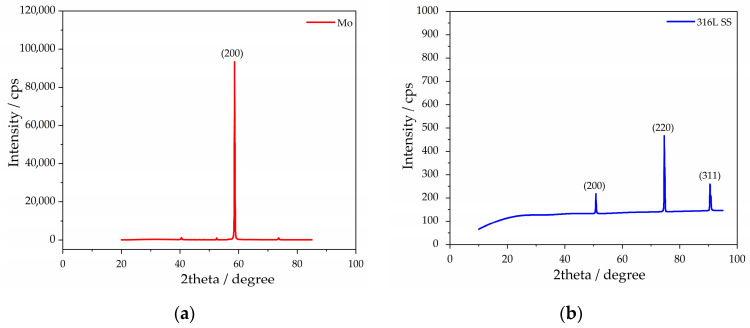
The XRD patterns of cathodes: (**a**) mono-crystalline Mo and (**b**) poly-crystalline 316L SS.

Figure 5 shows the XRD patterns of the Mo (Figure 5a) and 316L SS (Figure 5b) cathodes. The predominant diffraction peak at 2*θ* of 58.6° was observed for Mo (JPDCS 89-5023), and it corresponds to the (200) crystal plane [37] of the body-centered cubic (BCC) lattice. The other peaks observed at 40.5, 52.5 and 73.6 were insignificant, clearly pointing out that the used Mo represents a monocrystal of (200) preferred orientation. In the case of 316L SS substrate, three diffraction peaks located at 2*θ* of 54.6°, 74.6° and 90.5° are attributed to the (200), (220) and (311) crystallographic planes of γ austenite phase of the face-centered cubic (FCC) lattice of 316L SS [38]. Unlike the Mo substrate, 316L SS represents the typical polycrystalline substrate.

### 3.2. Analyses of Morphology, Topography and Microstructure of Electrolytically Produced Cu Foils

#### 3.2.1. Morphological Analysis of TCFs

**Figure 6:**
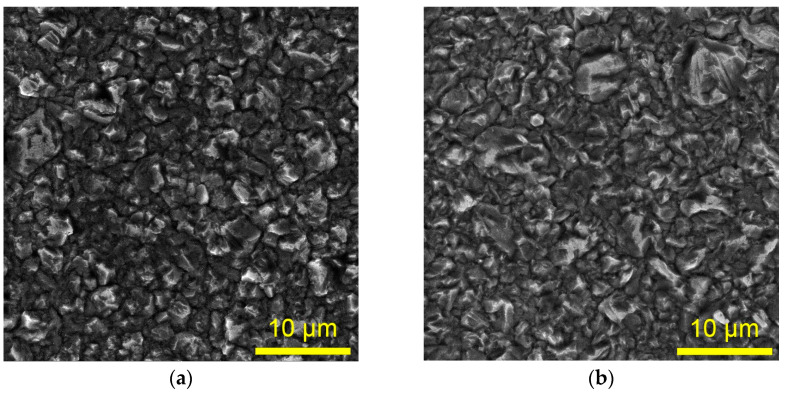

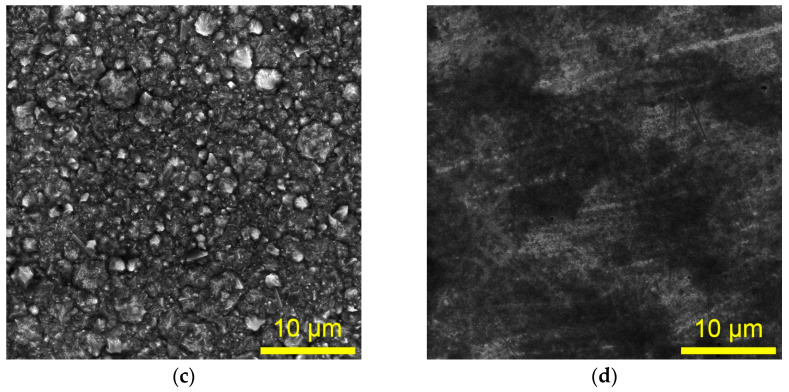
The morphology of Cu films obtained by the ED processes on the Mo cathode from: (**a**) solution I, (**b**) solution II, (**c**) solution III, and (**d**) solution IV. Magnification: ×5000.

**Figure 7:**
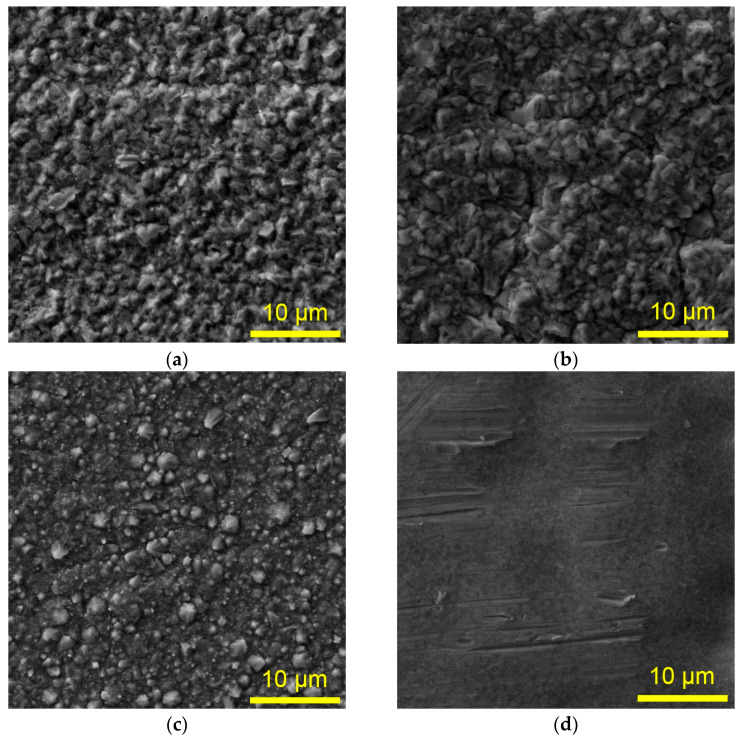
The morphology of Cu films obtained by the ED processes on the 316L SS cathode from: (**a**) solution I, (**b**) solution II, (**c**) solution III, and (**d**) solution IV. Magnification: ×5000.

The surface morphologies of Cu electrodeposited from the different solutions on the Mo and 316L SS cathodes are shown in Figure 6 (Cu/Mo) and Figure 7 (Cu/316L SS).

The first look at them shows that there is not any respectable effect of the substrate type on the morphology of Cu films, because it is mainly determined by the parameters of the electrodeposition, such as the composition of the electroplating solution and the current density, but not the cathode type. The microcrystalline (mc) films, which are mutually very similar, were obtained by the electrodeposition from solution I (the additive free solution) and solution II (the chloride ions are added to the sulfate solution): Figure 6a,b—Mo, and Figure 7a,b—316L SS. They consisted of grains with mostly uniform micro-sized dimensions. The addition of PEG 6000 in the sulfate solution with included chloride ions (solution III) caused non-uniformity in the Cu films on both substrates, as seen from Figure 6c and Figure 7c. The wide range of grain sizes observed at Cu films can mainly be attributed to the adsorption of large PEG 6000 molecules during the ED processing [20]. The dramatic change in the appearance of Cu films was obtained by the addition of MPSA in the solution with the chloride ions/PEG 6000 molecules (solution IV), Figure 6d and Figure 7d. The produced films received a mirror-bright appearance and it was established that they consisted of the atomically smooth parts of the surface [39]. The dimensions of features observed on these films were much smaller than in all other films (please see figures below).

#### 3.2.2. Analysis of Topography of Cu Films

**Figure 8:**
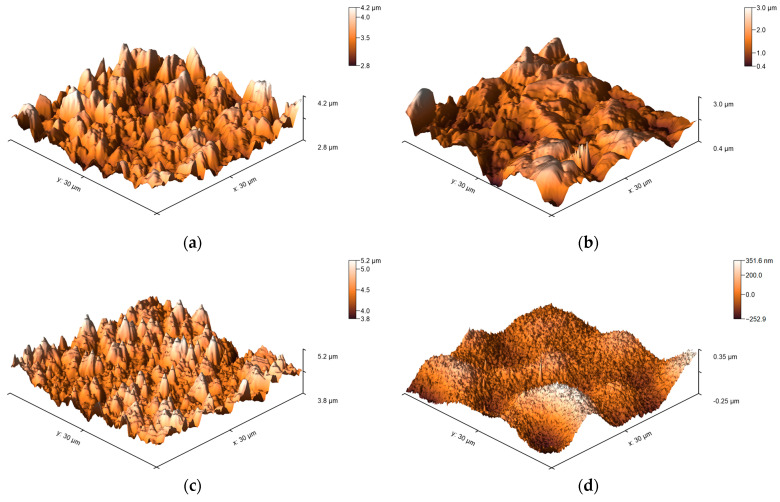
The topography of Cu films obtained by the ED processes on the Mo cathode from: (**a**) solution I, (**b**) solution II, (**c**) solution III, and (**d**) solution IV. Scan size: (30 × 30) μm^2^ [41].

**Figure 9:**
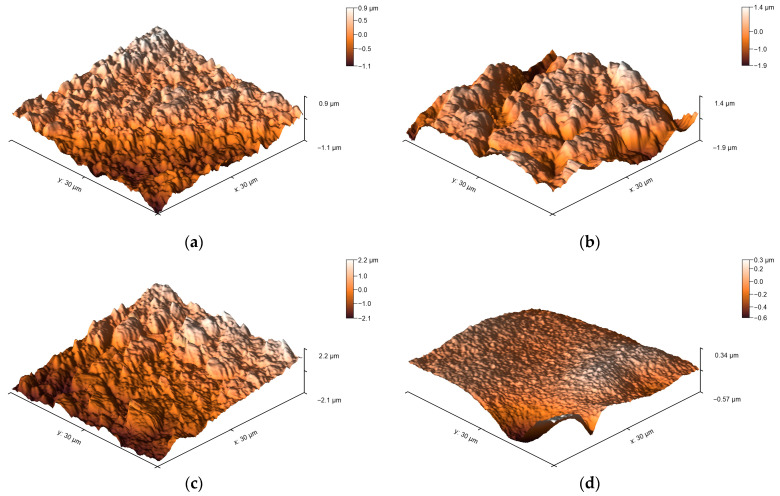
The topography of Cu films obtained by the ED processes on the 316L SS cathode from: (**a**) solution I, (**b**) solution II, (**c**) solution III, and (**d**) solution IV. Scan size: (30 × 30) μm^2^.

The surface topographies of Cu films electrodeposited from the different solutions on the Mo and 316L SS cathodes are shown in Figure 8 (Cu/Mo) and Figure 9 (Cu/316L SS). The values of the *S*_a_ surface roughness parameter [40] obtained for different Cu films are given in Table 2. The grain size was estimated by the AFM technique with the help of accompanying software. It is done by a determination of the grain height distribution (*h*_av_—average and *h*_max_—maximal). The histograms derived from the corresponding AFM images are included in the Supplementary Material as Figures S1 and S2, and the calculated average and maximal grain height size values have been added to the manuscript in Table 2.

It can be seen from Table 2 that Cu films electrodeposited on the 316L SS cathode are significantly rougher than Cu films electrodeposited on the Mo cathode for all used solutions. As an alloy, polycrystalline 316LSS contains numerous irregularities and boundaries among grains compared to the Mo monocrystal. A strong roughening effect is especially seen by the ED process from solution III on a relatively rough Mo substrate, and it can be attributed to the “additive competition” mechanism of the additives in super conformal Cu electrodeposition [42].

AFM analysis of the grain height distribution of Cu films strongly depended on both the cathode material and the electrolyte composition. Copper films electrodeposited on Mo exhibited lower average and maximum grain heights (*h*_av_ = 0.338–1.048 μm; *h*_max_ = 0.790–2.528 μm) than those obtained on 316L SS, confirming smoother and more uniform surfaces produced on Mo. In contrast, Cu films grown on 316L SS showed significantly higher values (*h*_av_ up to 1.941 μm; *h*_max_ up to 3.343 μm), reflecting coarser morphologies and pronounced surface relief. These differences highlight the decisive role of the type of cathode (Mo, BCC vs. 316L, FCC) in modulating additive–substrate interactions. While Mo promotes finer, more compact grain structures, 316L favors rougher growth, which may directly influence the adhesion and delamination behavior of the resulting Cu films.

#### 3.2.3. Structural Analysis of Cu/Mo and Cu/316L SS Films

**Figure 10:**
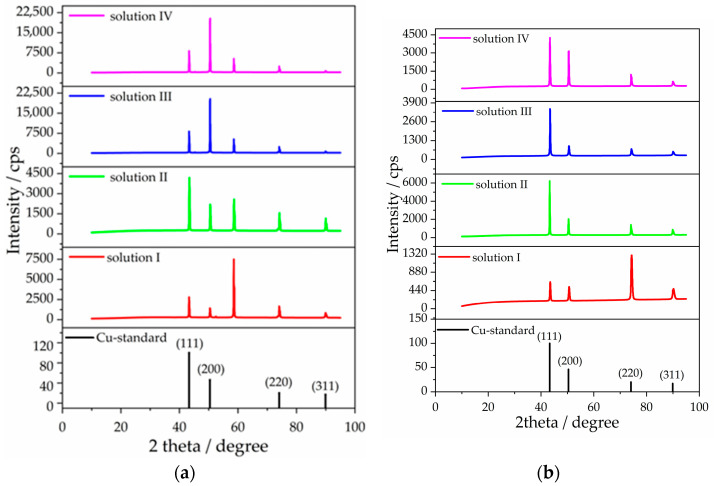
The XRD patterns of Cu films electrodeposited on two cathodes: (**a**) monocrystalline Mo and (**b**) polycrystalline 316L SS.

Figure 10 shows the XRD patterns of the Cu/Mo (Figure 10a) and Cu/316L SS (Figure 10b) films obtained from the solutions of the different composition. The diffraction intensities located at 2*θ* of 43.3°, 50.4°, 74.1°, and 89.9° are attributed to (111), (200), (220) and (311) crystal planes of the FCC (face-centered cubic) lattice of Cu. The different ratios of the diffraction peaks indicate the strong effect of a composition of the electroplating solutions on the crystal growth.

**Figure 11:**
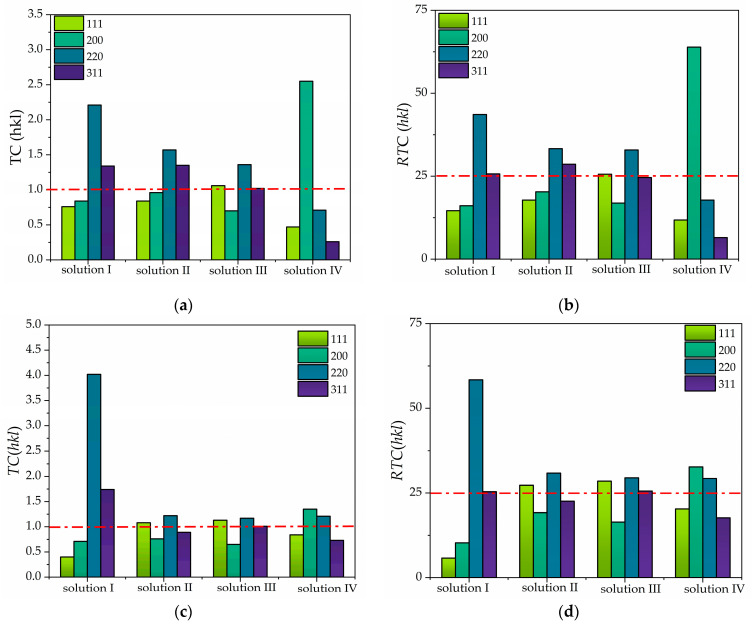
The histograms of texture coefficients and relative texture coefficients of Cu films electrodeposited on two cathodes: (**a**) Cu/Mo: *TC*(*hkl*), (**b**) Cu/Mo: *RTC*(*hkl*), (**c**) Cu/316LSS: *TC*(*hkl*), and (**d**) Cu/316L SS: *RTC*(*hkl*). The values above the red lines indicate the existence of the preferred orientation.

Therefore, a procedure based on a determination of texture coefficients (*TC*(*hkl*)) and relative texture coefficients (*RTC*(*hkl*)) was applied to estimate the preferred orientation of different Cu films. The coefficients calculated according to a procedure described in the Supplementary Materials (S2.4) [43] are presented in the form of histograms and given in Figure 11a and b for Cu/Mo—Figure 11a for *TC*(*hkl*) and Figure 11b for *RTC*(*hkl*), and in Figure 11c and Figure 11d for Cu/316L SS—Figure 11c for *TC*(*hkl*) and Figure 11d for *RTC*(*hkl*).

The Cu films electrodeposited on both substrates from the additive free solution (solution I) exhibited the (220) (311) preferred orientation. The addition of the chloride ions (solution II), as well as PEG in the solution with the added chloride ions (solution III) also promoted the preferred orientation in the (111) crystal plane. The PEG molecules in collaboration with the chloride ions suppress the electrodeposition process [21], resulting in the development of the (111) crystal plane with the lowest surface energy [44] as preferentially oriented. The top brightener, MPSA, was added to the solution with already-included chloride ions and PEG molecules (solution IV) activating the electrodeposition process [21], and this strongly modifies the crystal growth favoring the development of the preferred orientation in the (200) crystal plane. The type of substrates had the largest effect on the preferred orientation of mirror-bright Cu films, pointing the strong (200) for Cu/Mo and (200) (220) preferred orientation for Cu/316L SS films.

### 3.3. Analyses of Mechanical Properties of Cu Films Electrodeposited on Mo and 316L SS Cathodes

#### 3.3.1. Strengthening Mechanism of Cu Films

Based on the XRD data, the average crystallite size was calculated using the Williams–Hall method [45], and the calculated values are presented in Table 3.

The dependencies of the crystallite size on the solution composition observed for both Cu/Mo and Cu/316L SS systems make a profound impact on their mechanical performances. The tensile strength of the films is strongly governed by the Hall–Petch relationship, as this is the basic relationship which relates the size of the crystallites to the tensile strength of the film [46]. The dependence of the tensile strength *σ*(MPa) on the inverse square root of the grain for the crystallite size *d* (μm) is given by Equation (1) [46]:

(1) $$\sigma =64.05\cdot d^{-0.5}+184.96$$

**Figure 12:**
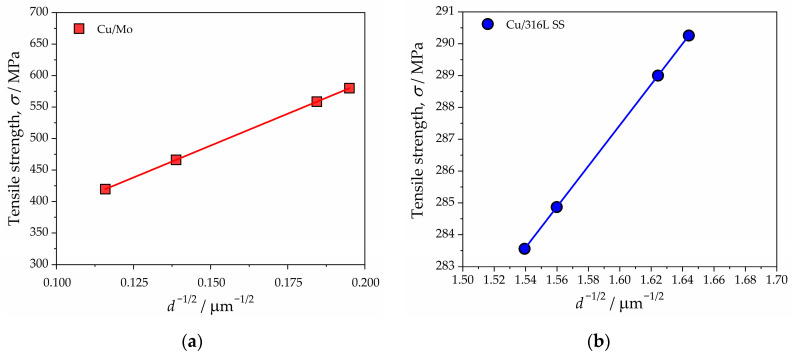
Hall–Petch relation (connection between the tensile strength and inverse square root of grain size) for Cu films electrodeposited on: (**a**) Mo, and (**b**) 316L SS cathodes.

The obtained fitted dependencies are shown in Figure 12a for Cu/Mo and Figure 12b for Cu/316L SS films.

The mechanical behavior of Cu films significantly depends on the crystallite size, due to the way that dislocations in a film interact with the grain boundaries. The decrease in the crystallite size causes a rise in the tensile strength in the film, as shown in Figure 12. As the copper crystallites in the films produced on the Mo were smaller than those produced on 316L SS cathodes, the tensile strengths of the Cu films on Mo were larger than those on 316L SS cathodes.

#### 3.3.2. Cyclic Bending Test—Delamination Resistance of Cu Films and Adhesion Properties

The cycling bending test is based on evaluating how flexible Cu films perform under repeated bending stresses, and the higher value of *n*_c_ indicates that the adhesion of a film is better [47]. The adhesion test results obtained by this test given in Table 4 showed that the Cu films produced on the Mo substrate have better adhesion properties than those obtained on the 316L SS substrate. The three series of the films done under identical conditions were tested and the number of cycles was the same in all cases. Also, the Cu films electrodeposited from solution III on the Mo cathode achieved the highest critical cycle number (10.5), and hence, the best adhesion, while the Cu film electrodeposited from solution IV on the 316L SS substrate had the lowest value of the critical cycle numbers (1.5) and hence, the worst adhesion. This indicates that adhesion strength is strongly influenced by both the composition of the solution and the type of substrates.

Generally, the worst adhesion exhibited mirror bright, very smooth Cu films obtained from the solution IV on both substrates. The obtained results were in good agreement with the morphological and structural characteristics of the films.

Further analysis of the composite system of the copper film electrodeposited on Mo was performed using cross-sectional/mapping analyses, and determination of the chemical composition of the copper film and thickness.

### 3.4. Morphologies and Elemental Analysis by EDS/Mapping of Cross-Section of Cu/Mo Films

**Figure 13:**
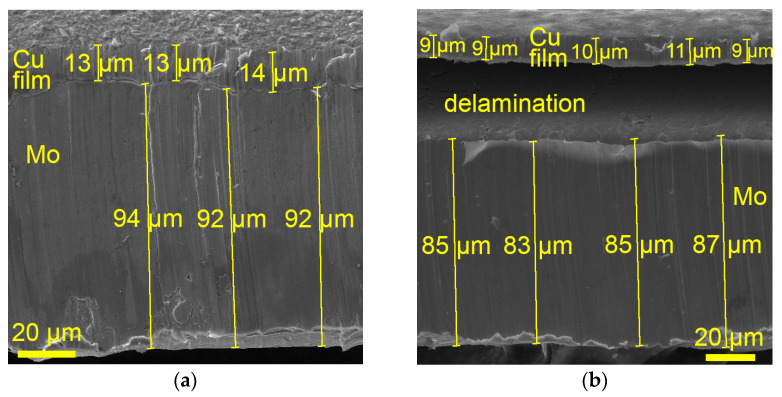
Cross-sectional FE-SEM images of Cu films prepared on Mo cathode from: (**a**) solution I and (**b**) solution IV.

Elemental analysis of the cross-sections of Cu/Mo films is performed to evaluate the film thickness, interface diffusion, stoichiometry, contamination, and degree of delamination. As an example, the cross-sections of Cu films electrodeposited on the Mo cathode from solution I and solution IV are analyzed and shown in Figure 13 (solution I/Figure 13a, and solution IV/Figure 13b).

In spite of the same electrodeposition time, the composition of the solution had a strong influence on the thickness of the film. The thickness of the film obtained from additive free solution (solution I) was 13.3 μm, while the mirror-bright Cu film obtained from the solution with all three additives included (solution IV) was 9.6 μm. Certainly, this significant difference of about 28% is a consequence of a complex mechanism of Cu electrodeposition in the presence of a combination of these additives, based on the suppression/activation of the electrodeposition process [5,6,9,41]. The model of “local perforation” is usually proposed to explain the synergistic effect of these additives [5,39].

**Figure 14:**
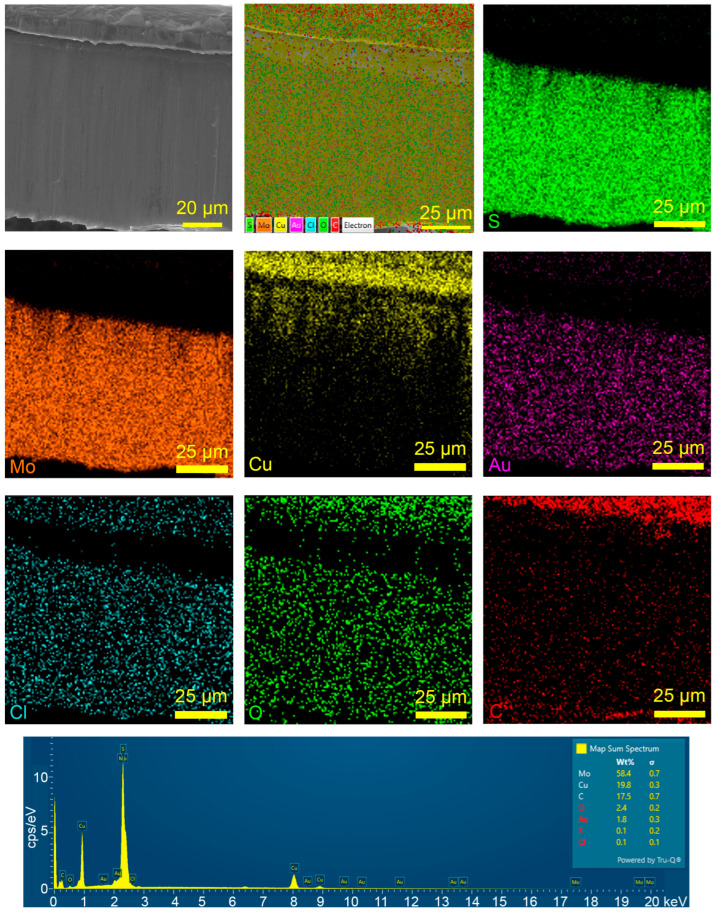
Cross-sectional FE-SEM/EDS images of Cu film prepared on a Mo cathode from solution IV with mapping analysis.

The cross-sectional EDS/mapping of Cu films is done to observe the chemical composition of the resulting copper films. The mapping of the film electrodeposited from electrolyte IV is shown in Figure 14, while Table 5 presents the results of the chemical analysis for the Cu/Mo samples. The uniform distribution of Mo, Cu, Au, C, O, Cl, and S atoms is observed through the interior of this film, confirming the successful incorporation elements from additives in the film.

The procedure for the preparation of the sample for EDS analysis is a reason for relatively high C content. It requires evaporation to provide a conductive layer for the cross-section analysis. The origin of Cl and S in the cross-section of the films is the additives added to the sulfate solutions, while O probably originates from oxide formed in the air before EDS analysis.

### 3.5. Wettability of Cu Films

Wettability is very important property of metal films for the application of films in microelectronics and energy storage systems. For these applications, the films should be resistant to wetting by different fluids. The measurement of contact angles is one of the ways to estimate wettability.

**Figure 15:**
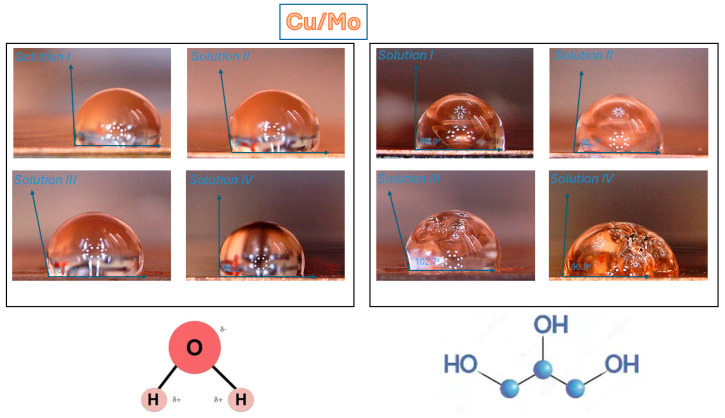
The appearance of water and glycol droplets on Cu films electrodeposited on Mo substrate from solutions I, II, III, and IV.

**Figure 16:**
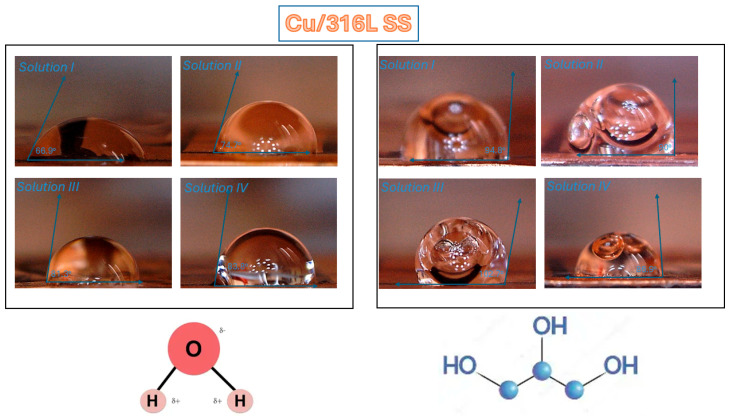
The appearance of water and glycol droplets on Cu films electrodeposited on 316L SS substrate from solutions I, II, III, and IV.

**Figure 17:**
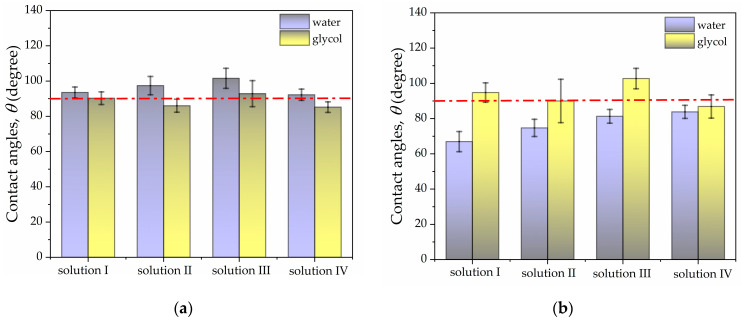
The histograms of measured values of water and glycol contact angles on the Cu films electrodeposited on: (**a**) Mo, and (**b**) 316L SS cathodes from four solutions according to the sessile drop method. The red dashed line indicates the boundary between the hydrophilic and hydrophobic properties of the surface.

The static water and glycol contact angles (*θ*_1_ and *θ*_2_) are defined as the angles formed between the solid surface of a film and the chosen fluids. When the water contact angle is lower than 90°, the water droplet spreads easily, indicating the hydrophilic behavior of a film surface [48]. Conversely, contact angles higher than 90° correspond to the hydrophobic behavior of a film [49]. In addition to the distilled water, glycol as a more viscous component was selected to estimate the wettability properties [50,51]. Figure 15 and Figure 16 illustrate the appearance and shape of glycol and water droplets formed on Cu/Mo (Figure 15) and Cu/316L SS (Figure 16) films electrodeposited from all four solutions. The measured contact angles are summarized at the histograms given in Figure 17a (for Cu/Mo) and Figure 17b (for Cu/316 SS).

Regarding water as a fluid, Cu/Mo films produced from all four solutions exhibited a hydrophobic characteristic, while Cu/316L SS films had a hydrophilic characteristic. The Cu films electrodeposited on the Mo cathode have lower glycol contact angles than water, and their behavior is at a boundary between hydrophilic and hydrophobic characteristics. This was expected because glycol is a less polar liquid than water, with weaker hydrogen bonding. Glycol can interact more favorably with the Cu film surface, lowering the contact angle in comparison with water. It can be seen from the histograms shown in Figure 17 that Cu/316L SS films (Figure 17b) have a more hydrophilic nature than Cu/Mo films (Figure 17a). It is consistent with the Wenzel relation [51], which predicts that a naturally hydrophilic surface will show lower hydrophilicity, i.e., it will exhibit a higher contact angle if its surface roughness is reduced. It can easily be confirmed by a comparison of the roughness values of Cu/316L SS films electrodeposited from solution I (337.2 nm) and electrolyte IV (85 nm) and the corresponding water contact angle values of 66 ± 5.7° (solution I) and 84 ± 3.7 ° (solution IV).

For hydrophobic surfaces such as Cu/Mo, the increased roughness (Table 2) amplifies hydrophobicity (from 92° to 101°), causing droplets to bead up more strongly. On these surfaces, the influence of the surface chemistry and changes in the chemical composition of Cu films due to the incorporation of elements from additives is dominant. It can be seen from Figure 17 that the contact angles of Cu/316L SS films were larger than those for Cu/Mo films. This can be attributed to the passive oxide layer formation on stainless-steel, which alters the surface energy of electrodeposited Cu by the introduction of the polar functional groups at the interface [52]. These oxides are absent in the case of Mo, making the Cu film less polar and more hydrophobic at this substrate.

Based on the measured contact angles of the water and glycol, the theoretical value of the Laplace pressure was calculated. This pressure is important because a higher Young–Laplace pressure indicates film stresses, which can lead to cracking or peeling of the film from the substrate. It provides a correlation between the surface tension and curvature, as well as the stability and quality of TCFs. The Laplace pressure for different Cu films was calculated using the Yang–Laplace Equation (2) [35]:

(2) $$\Delta p=p-p_{0}\approx \frac{2\cdot \gamma \cdot \cos(\theta )}{R}$$

where *γ* is the surface tension of the water or glycol (*γ*_H2O_ = 72.8 N/m, γ_glycol_ = 64 N/m), *θ* is the contact angle (water or glycol), and *R* is the equivalent spherical radius of the liquid droplets. If the droplet is approximated as a perfect sphere, *R* can be determined from its volume. The equivalent spherical radius was calculated as 0.0010609 m. The values of the Yang–Laplace pressure (Δ*p*) for the Cu films are given in Table 6.

From Table 6, it can be seen that the substrate type, solution chemistry (electrolytes), and test liquid all significantly affect the capillary pressure. Cu films electrodeposited from solution III produced the highest Δ*p* for Mo in water (27.27 kPa) and for 316L SS in glycol (26.57 kPa), highlighting the strong influence of solution composition. The greatest capillary effects were observed in the mentioned films. As additives change the contact angle (surface energy) and shape or curvature, and therefore the Young–Laplace pressure, this explains why films with additives have different thicknesses and appearances.

## 4. Discussion

The influence of a combination of organic/nonorganic additives added as suppressor/activator of the ED process in the Cu-based sulfate solution and the type of cathode material on morphology, structure, adhesion resistance, chemical composition, wettability, and Yang–Laplace pressure of galvanostatically produced Cu films has been investigated and analyzed. The following electroplating solutions were used for synthesizing Cu films: a basic sulfate electrolyte (solution I), an electrolyte with added chloride ions (solution II), a combination of chloride ions and PEG (solution III), and a combination of all the additives (chloride ions + PEG 6000 + MPSA), solution (IV).

Since the goal of the work was to produce thin copper foils (TCFs) that were suitable for use as current collectors, it was necessary to produce Cu films which easily delaminate from the cathode. For that reason, the choice of cathode material is very important, and Mo and 316L SS were tested for this purpose. The manual delamination of the Cu film is not desirable because a partial separation of the TCFs from the cathode or film cracking may occur [53]. The laboratory prototype device constructed for evaluating the adhesiveness of the films (Figure 1, Figure 2 and Figure 3) is simultaneously used for estimating the Cu film delamination from the cathode.

During the bending of the metal film–substrate system, the changes occur on both the film surface and the film–substrate interface, due to increased stress [54]. Consequently, the metallic film detaches from the substrate after a certain critical number of bending cycles, *n*_c_, and the values obtained for Cu films electrodeposited on Mo and 316L SS cathodes are given in Table 4. The concept of this device was based on an investigation of Zhu et al. [47], who used a simple steel clamp to bend coated substrates and count cycles until delamination occurred. Unlike their device, which was not automated and can cause an uncontrolled bending force, this device is automatic with defined force and speed of bending.

The smallest critical number of cycles, *n*_c_, i.e., the easiest delamination or the worst adhesion of Cu film, is obtained for the mirror-bright Cu film obtained from the solution IV on both cathodes (2 on Mo, and 1.5 on 316L SS). In contrary, the *n*_c_ values obtained for Cu film electrodeposited from the same solution on the brass cathode [36] were incomparably larger than those obtained on Mo and 316L SS. This clearly points out that a selection of the cathodic material plays a crucial role in fabricating Cu foils. A similar conclusion can be derived by the comparison of *n*_c_ values for the Cu films electrodeposited from solution I on different substrates. The *n*_c_ values of four for the film on Mo, and two for that on 316L SS (Table 4) were significantly lower than the values obtained from the Cu cathode for the films of the same thickness under different stirring conditions (*n*_c_ in (114 ÷ 130) range) [36].

The best adhesion (the most difficult) delamination of the Cu film from the cathode is obtained for the Cu film electrodeposited from solution III (PEG + Cl^−^ ions) on Mo with *n*_c_ ≈ 10.5. These additives produced the fine-grained films with grains of spherical form and roughness that were larger than other films. The statement agrees with the previous results of adhesion testing using the Vickers method for the same electrolyte composition [20]. PEG’s hydrophilic and antistatic properties, along with its high surface energy, are responsible for an enhanced adhesion of these films.

The validation of the bending adhesion test method was done with a standard test method with tape, known as a “scratch-tape adhesion test”. The details are given in the Supplementary Material (see S2.6) [55]. The use of this test confirmed the weak adhesion of the Cu films electrodeposited on these substrates.

The correlation between the crystallography and adhesion of the Cu films electrodeposited on Mo substrate showed that adhesion is weak for the Cu film from solution IV, where the strong (200) preferred orientation is dominant, and is better for the films obtained from solutions I and II, which are governed by (220) and (311) preferred orientations (Figure 11). The better adhesion of Cu/Mo compared to Cu/316L SS films may result from larger reduction in the Cu crystallite size in Cu/Mo films (Table 3) caused by the crystallographic compatibility between the film and the cathode. Molybdenum is mono-crystalline (Figure 5a) and has a spatial lattice that better matches the cubic lattice of Cu, resulting in lower lattice mismatch and lower stress at the interface [56]. This leads to better adhesion, since the crystallographic match facilitates diffusion bonding and reduces defects. An alloy such as 316L SS has a more complex and heterogeneous polycrystalline structure (Figure 5b), making it more difficult for the cubic lattice of copper to fit into heterogeneous structure of steel. This results in a stress concentration at the grain boundaries and interfaces, which weakens the adhesion properties of the film [57]. Also, the Mo cathode has a higher surface roughness than steel, which can be an additional reason for the better adhesion of Cu on Mo than on steel substrates (Figure 4). Although the single crystal was expected to have low roughness, in this case, the substrate was prepared by grinding, which increased its initial roughness. The roughness of the Mo substrate is increased by about three times compared to the steel substrate (Figure 4). However, for the case where film delamination is desired, stress generation during the bending test is beneficial.

The morphology of Cu films is primarily determined by electrodeposition parameters, such as the solution composition and current density, rather than by cathode type. This is a reason for similar surface morphologies being obtained on the Mo and 316L SS substrates. The roughness of the films is determined by the state of the cathode surface used for the electrodeposition, but the films of different roughness obtained on different substrates under the same conditions have the same surface morphology at the macro level, which is shown in this study. Simultaneously, the type of cathodic material with crystallographic planes affects the development of the preferred orientation of the films, producing the films with the same morphology, but with a different crystal structure. The microcrystalline Cu films with uniform grains were obtained from an additive-free sulfate solution (I) and chloride-containing solution (II). Adding PEG 6000 (solution III) produced non-uniform films with a wide grain size distribution, due to PEG adsorption. A striking change occurred with the MPSA addition (solution IV), yielding mirror-bright films composed of atomically smooth, nano-dimensional grains.

The easy delamination of the mirror-bright Cu film obtained from solution IV from the Mo cathode was also confirmed by the cross-section analysis of this film (Figure 13b). Simultaneously, the fine-grained Cu film electrodeposited from solution I without additives (Figure 13a) did not show a delamination during the analysis of the cross-section. This is in accordance with the results given in Table 4, since the adhesion of the Cu film obtained from the solution without additives is twice as large as that from solution IV. The thickness of the electrodeposited Cu film changed depending on the composition of the solution. When all additives were added to the solution (solution IV), the film thickness is 9.6 μm, and this was smaller than that obtained from the pure sulfate solution (solution I: 13.3 μm). It is generally known that electrodeposition is controlled by the diffusion of Cu^2+^ ions and the current density [58]. The Cu film grows rapidly from solution I; the grains are coarse and the surface is rough (Table 2) (Figure 6a and Figure 8a). The combination of all additives (solution IV) creates a strong inhibition, because PEG covers the surface and MPSA binds to Cu^2+^ and additionally slows down the reduction [59]. The result of this process is a thinner Cu film with good uniformity.

The EDS mapping did not show the presence of chlorine in Cu films deposited from solution III, but detected it in samples deposited from solution II and solution IV. In solution III (PEG + Cl^−^), chloride is not detected because PEG stabilizes chloride only as a transient surface suppressor. Cl^−^ adsorbs during deposition but does not remain incorporated in the copper lattice, so it is absent in the cross-section analysis. In solution II (only Cl^−^) and solution IV (PEG + Cl^−^ + MPSA), chloride is detected. With MPSA, the thiol–sulfonic groups form stable complexes with Cl^−^ at the cathode, allowing some chloride to remain bound and be incorporated into the growing film [59]. This is why chloride is not detected in the cross-section of the Cu film with PEG + Cl^−^ (solution III), while in the system with MPSA, chloride remains bound and is analytically detectable. The incorporation of sulfur into Cu films electrodeposited from solution IV is obvious, and it originates from the MPSA additive, i.e., thiol and sulfonic groups. Hence, depending on the type of additive, the strong influence on morphology, topography, crystal structure, and on the mechanical properties (adhesion) of the Cu films were observed.

MPSA contains both a thiol (–SH) group and a sulfonic acid group [60]. The thiol group has a strong affinity for copper surfaces, meaning that MPSA molecules adsorb readily during the electrodeposition. This adsorption blocks active growth sites and slows down Cu ion reduction. While additives like PEG 6000 and Cl^−^ promote finer, denser films with a larger number of grains which simultaneously increase the number of boundaries among grains, MPSA causes a smoothness effect on the film, decreasing the number of boundaries among grains. The sulfonic group introduces repulsion between the adsorbed molecules, which can hinder the dense packing of Cu atoms. The numerous studies claim that MPSA has a catalytic function [61], significantly reduces the inhibiting properties of PEG [62], and decreases the adhesion between the Cu film and substrate compared to PEG or Cl^−^ alone [20].

**Scheme 2:**
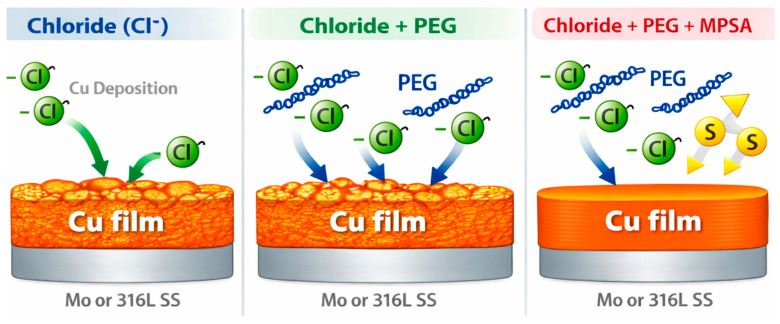
Schematic representation of additive effects during electrodeposition process on Mo and 316L SS substrates.

A schematic presentation of the effect of used additives on Cu electrodeposition processes is presented in Scheme 2.

Any special contribution of the type of cathode on the mechanism of Cu electrodeposition in the presence of additives cannot be established. The action of these additives can be summarized as follows: the adsorbed chloride layer is formed by the addition of the chloride ions into the basic sulfate solution [63]. Polyethylene glycol (PEG) added into the solution with the chloride ions already added inhibits the electrodeposition process through the formation of a barrier onto the electrode surface [64]. In this way, PEG in combination with the chloride ions acts as suppressor of the electrodeposition process. Finally, MPSA added into the solution with the included chloride ions and PEG activates the electrodeposition process through a disruption of the inhibiting layer, making the penetration of Cu(II) ions through this layer possible.

The Laplace pressure has a direct effect on the adhesion of the thin film to the substrate [35,51]. The calculated values of the Laplace pressure for Cu films electrodeposited from solutions I, II, III, and IV on the Mo substrate are 8.4, 17.6, 27.3 and 5.30 kPa, and for those on 316L SS: 53.34, 17.95, 20,65, and 14.66 kPa, respectively. The curvature radius of the droplet (Figure 15 and Figure 16) and the large Laplace pressure indicate the appearance of micro- and nano-pores in the film. For example, the Cu film electrodeposited from electrolyte III has the highest Laplace pressure, a smaller effective radius of the droplet, and it is more difficult to wet. This statement is also consistent with the roughness parameters for the examined Cu films.

## 5. Conclusions

Molybdenum (Mo) and stainless-steel (316L SS) were analyzed as substrates in Cu ED processes from acid sulfate solutions with the final aim of obtaining Cu in the form of foil. To realize this task, different solutions were analyzed, including the basic sulfate solution and solutions with included suppressor/activator additives, which enabled the electrochemical deposition of Cu films from microcrystalline with different degrees of fine-grained mirror-bright nanocrystalline films. The adhesion of Cu films to the cathode was examined by a lab-made bending test machine made by an application of additive technology. As a parameter for the estimation of the adhesion characteristics of the films, this device uses a critical cycle number that defines a minimal number of cycles, leading to a delamination of the film from the cathode.

The use of various additives added individually or in a combination in the acidic sulfate solution did not only affect the morphology of the electrodeposited Cu, but also the structure, the tensile strength and the wettability.

The fastest delamination of the Cu films from the substrate (the weakest adhesion) was observed for the nanocrystalline mirror-bright films obtained with an addition of all three additives (chloride ions, polyethylene glycol 6000 and 3–Mercapto–1–propanesulfonic acid). The delamination of the films slowed down, i.e., the critical number of cycles leading to the delamination increased when the microcrystalline films were analyzed.

The values of the critical number of cycles were significantly lower than the values obtained for the Cu films that were electrodeposited on the native Cu cathode, clearly pointing out the crucial role of the cathode type in the production of Cu foil.

Based on the easy and fast delamination of the Cu films from Mo and 316L SS cathodes, it follows that these substrates can successfully be used for fabricating Cu foils.

## Figures and Tables

**Table 1:** Electrodeposition parameters and a composition of solutions.

	Composition of Electroplating Solution *	Solution Label	The Working Conditions
1.	CuSO_4_ × 5 H_2_O + H_2_SO_4_	Solution I	
2.	CuSO_4_ × 5 H_2_O + H_2_SO_4_ + NaCl	Solution II	
3.	CuSO_4_ × 5 H_2_O + H_2_SO_4_ + NaCl + PEG 6000 (polyethylene glycol)	Solution III	Current density, *j*, *j* = 60 mA cm^−2^ Temperature, *t*, *t* = 22.0 ± 0.5 °C
4.	CuSO_4_ × 5 H_2_O + H_2_SO_4_ + NaCl + PEG (polyethylene glycol) 6000 + 3–Mercapto–1–propanesulfonic acid (MPSA)	Solution IV	The electrodeposition time: 7.5 min Solution stirring: 100 rpm

* *c*(CuSO_4_ × 5 H_2_O) = 240 g dm^−3^; *c*(H_2_SO_4_) = 60 g dm^−3^; *c*(NaCl) = 0.124 g dm^−3^; *c*(PEG 6000) = 1 g dm^−3^; and *c*(MPSA) = 0.0015 g dm^−3^.

**Table 2:** The values of the surface arithmetic average roughness parameter (*S*_a_) in nm, for Cu films synthesized on Mo and 316L SS cathodes from different solutions and the values of the grain height distribution calculated by use of AFM software (Gwyddion, v. 2.61).

Cathode/Solution⟹	Solution I	Solution II	Solution III	Solution IV
Mo				
*S*_a_	186.3	146.7	343.7	71.98
*h*_av_	0.585	0.707	1.048	0.338
*h*_max_	1.119	1.397	2.528	0.790
316L SS				
*S*_a_	337.2	249.9	386	85.0
*h*_av_	0.593	1.078	1.941	0.571
*h*_max_	1.406	2.352	3.343	0.909

**Table 3:** The values of the crystallite size (*d*) in nm, obtained for Cu films on Mo and 316L SS cathodes from the different solutions.

Cathode/Solution⟹	Solution I	Solution II	Solution III	Solution IV
The value of the grain size (*d*) in nm				
Mo	74.5	51.9	29.4	26.3
316L SS	422	411	379	370

**Table 4:** Results of cyclic bending test applied obtained on the lab-made machine for Cu films synthesized from the different solutions on Mo and 316L SS cathodes.

Cathode/Solution⟹	Solution I	Solution II	Solution III	Solution IV
*n_c_*				
Mo	4	5.5	10.5	2
316L SS	3	2.5	5	1.5

**Table 5:** Results of the elemental analysis of Cu films synthesized from the solutions without/with additives on Mo substrate.

Elements/Solution⟹	Solution I	Solution II	Solution III	Solution IV
Atomic %				
Mo	32.42	40.71	44.29	23.93
Cu	17.47	16.53	24.09	12.24
Au	0.58	0.69	0.67	0.35
C	42.21	35.52	25.62	57.32
O	7.32	6.37	5.33	5.90
Cl	/	0.19	/	0.08
S	/	/	/	0.17

**Table 6:** The value of the Yang–Laplace pressure (Δ*p*) in kPa, for Cu films on Mo and 316L SS cathodes synthesized from different solutions and tested with two liquids (water and glycol).

Cathode/Solution⟹	Solution I	Solution II	Solution III	Solution IV
water				
Mo	8.36	17.60	27.27	5.30
316L SS	53.34	17.95	20.65	14.66
glycol				
Mo	0.58	8.33	5.99	9.97
316L SS	10.09	0.21	26.57	6.61

## Data Availability

The original contributions presented in this study are included in the article/Supplementary Material. Further inquiries can be directed to the corresponding authors.

## Supplementary Materials

The following supporting information can be downloaded at: https://www.mdpi.com/article/10.3390/ma19091838/s1. Figure S1. The histograms of the height of grains distribution for Cu films obtained by the ED processes on the Mo cathode from: (a) solution I, (b) solution II, (c) solution III, and (d) solution IV. Figure S2. The histograms of the height of grains distribution for Cu films obtained by the ED processes on the 316L SS cathode from: (a) solution I, (b) solution II, (c) solution III, and (d) solution IV. Figure S3. The scratch-tape adhesion test: (a) Cu film on 316L SS cathode before delamination, (b) 316L SS cathode after pilling Cu film, and (c) delaminated Cu foil on the 3M tape. Table S1. Chemical composition of Mo and 316L SS substrates. Table S2. Texture calculations for the Cu/Mo films produced galvanostatically from solutions without/with additives (solution I, II, III, IV) at a current density of 60 mA cm^−2^. *R*—Intensity of the diffraction peak; *TC*—Texture Coefficient; *RTC*—Relative Texture Coefficient; *s*—Cu standard. Table S3. Texture calculations for the Cu/316L SS films produced galvanostatically from solutions without/with additives (solution I, II, III, IV) at a current density of 60 mA cm^−2^. *R*—Intensity of the diffraction peak; *TC*—Texture Coefficient; *RTC*—Relative Texture Coefficient; s—Cu standard.

## Abbreviations

The following abbreviations are used in this manuscript:

TCFs	Thin Copper Foils
CBCs	Current Battery Collectors
MPSA	3-Mercapto-1-propanesulfonic acid sodium salt
PEG	Polyethylene Glycol
Cl^−^	Chloride Ions
SEM	Scanning Electron Microscope
AFM	Atomic Force Microscope
XRD	X-ray Diffraction
CVD	Chemical Vapor Deposition
PVD	Physical Vapor Deposition
M-CCs	Metal-based Current Collectors
HAR	High Aspect Ratio Trenches
ED	Electrodeposition
