# Supplementary material for: Thin Copper Foils: From Electrodeposition Conditions to Adhesion Performances
Source: Materials (Basel). 2026 Apr 29;19(9):1838. doi: 10.3390/ma19091838 (PMC13164644; doi:10.3390/ma19091838)
Supplement: Supplementary file 1 [file materials-19-01838-s001.zip › materials-4275121-supplementary.pdf]

Thin Copper Foils: From Electrodeposition to Adhesion Performance

Ivana O. Mladenović[1,*], Željko Radovanović[2], Dana G. Vasiljević Radović[1], Rastko Vasilić[3], Miloš Vorkapić[1], Predrag Živković[4], and Nebojša D. Nikolić[1,*]

1 Institute of Chemistry, Technology and Metallurgy, University of Belgrade, Njegoševa 12, 11000 Belgrade, Serbia; ivana.mladenovic@ihtm.bg.ac.rs (I.O.M.), dana.vasiljevic@ihtm.bg.ac.rs (D.G.V.R.), milos.vorkapic@ihtm.bg.ac.rs (M.V.), nnikolic@ihtm.bg.ac.rs (N.D.N.)
2 Innovation Center of Faculty of Technology and Metallurgy LTD. in Belgrade, Belgrade, Serbia; zradovanovic@tmf.bg.ac.rs (Ž.R),
3 Faculty of Physics, University of Belgrade, Studentski Trg 12-16, 11000 Belgrade, Serbia; rastko.vasilic@ff.bg.ac.rs (R.V.)
4 Faculty of Technology and Metallurgy, University of Belgrade, Karnegijeva 4, 11000 Belgrade, Serbia; peca@tmf.bg.ac.rs (P.Ž.)
* Correspondence: ivana.mladenovic@ihtm.bg.ac.rs (I.O.M.); Tel.: + 381 11 263 6576 and nnikolic@ihtm.bg.ac.rs (N.D.N.); Tel.: + 381 11 337 03 90

Supplementary Material

S2.2. Materials and methods – additional information

The high-purity water, 18 MΩ·cm (Millipore,Burlington, MA, USA) and p.a. reagents were used for a preparation of electroplatings solutions.

The following procedure was applied for a preparation of Mo and 316L SS substrates:

a) Mo cathode with a purity > 99.9 wt. % , and 200 μm-thick was polished with SiC paper (1500 grits) and rinsed ultrasonically. After mechanical polishing, the specimens are cleaned by ultrasonic in an acetone bath, then dried and weighted.
b) the 316L SS cathode was degreased, polished, and rinsed. As a 316L SS cathode has a passive chromium oxide film, acid pickling in sulfuric acid (H_2SO_4) solution is applied, and rinsed in water and drying in N_2.
c) copper anodes was polished chemically in acid solution before electrodeposition (HNO_3:H_2O = 1:1 vol. %).

Table S1. Chemical composition of Mo and 316L SS substrates.

Mo / %			*316L SS / %*									
Mo	*O*	*Au*	*Fe*	*Cr*	*Mn*	*Ni*	*Mo*	*Si*	*P*	*S*	*N*	*C*
95.87	*4.12*	*0.01*	*62-70*	*16-18*	*2*	*10-13*	*2*	*1*	*0.04*	*0.015*	*0.11*	*0.03*

S2.3. The characterization of electrodeposited Cu films

a) the surface morphology and qualitative compositional analysis: it is used a scanning electron microscope (SEM), model FE-SEM, MIRA3 TESCAN XMLI, Oxford, UK. This device was equipped with Energy Dispersive X-ray Diffraction Spectroscopic Analyzer, EDS, model INCAx-act LN2 (Oxford Instruments, Oxford, UK), with the PentaFET® Precision and Aztec 4.3 software package (Oxford Instruments, Oxford, UK) [20-23].

b) the topography and roughness analysis: it is used an atomic force microscope (AFM): model Auto Probe CP Research; TM Microscopes–Veeco Instruments, Santa Barbara, CA, USA [20-23, 26-27]. Surface roughness parameter (S_a,) were calculated from mid-plane AFM images using Gwyddion 2.61 (Open-Source software, Czech Metrology Institute, Jihlava, Czechia) [40]. S_a represents the average height deviation across a surface essentially the standard deviation of the surface profile [20-23, 40].

c) structure: it is used a model Rigaku Ultima IV diffractometer, Rigaku Co. Ltd., Tokyo, Japan, in Bragg–Brentano geometry with CuKα radiation [20-23, 26-27]. The scanning rate of 2° min^{-1} in the 2θ ranges 20–85° for Mo, 20–95° for 316L SS, and 20–95° for Cu were used.

d) wettability of the Cu foils was determined via static contact angle measurements using the sessile drop technique. Droplets (5 µL) of deionized water (18 MΩ·cm) and glycol were deposited at five random positions on each sample. Images were taken 5 s after deposition with an optical microscope (Delta Optical Smart 5.0 MP Pro, Poland) [22, 23, 49] and analyzed using GIMP software (version 3.0.0.-1, free software of GNU General Public License as published by the Free Software Foundation). All tests were carried out at room conditions (25 °C, 50% RH).

S2.4. AFM analysis

Figure S1 represents the histograms of the grain height distribution of Cu films on Mo cathode electrodeposited from the solution I (Figure S1a), solution II (Figure S1b), solution III (Figure S1c), and solution IV (Figure S1d).

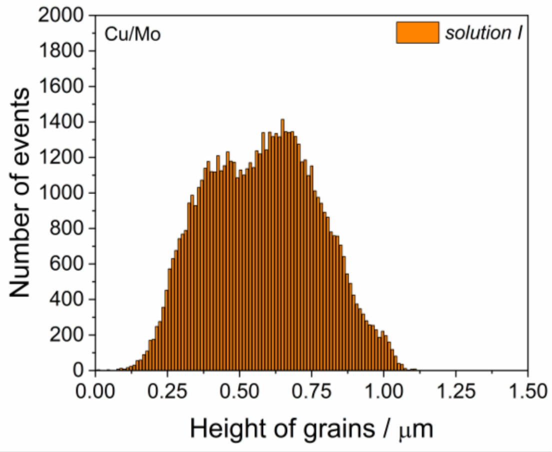

(**a**)

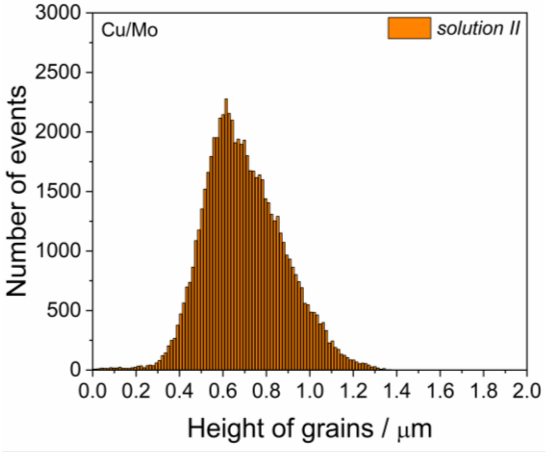

(**b**)

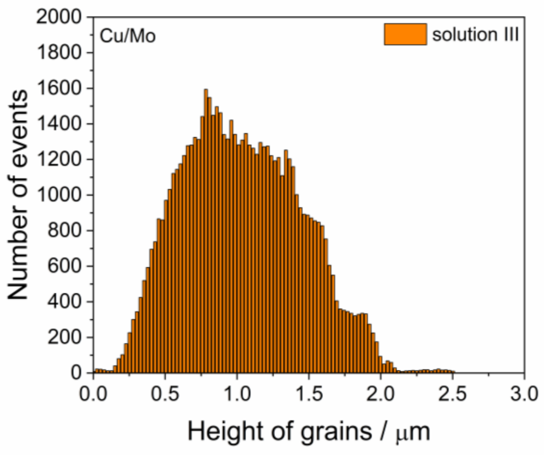

(**c**)

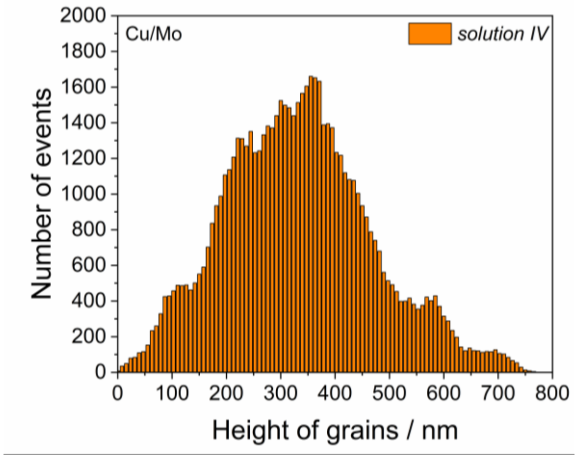

(**d**)

Figure S1. The histograms of the height of grains distribution for Cu films obtained by the ED processes on the Mo cathode from: (**a**) solution I, (**b**) solution II, (**c**) solution III, and (**d**) solution IV.

Figure S2 represents the histograms of the grain height distribution of Cu films on 316L SS cathode electrodeposited from the solution I (Figure S2a), solution II (Figure S2b), solution III (Figure S2c), and solution IV (Figure S2d).

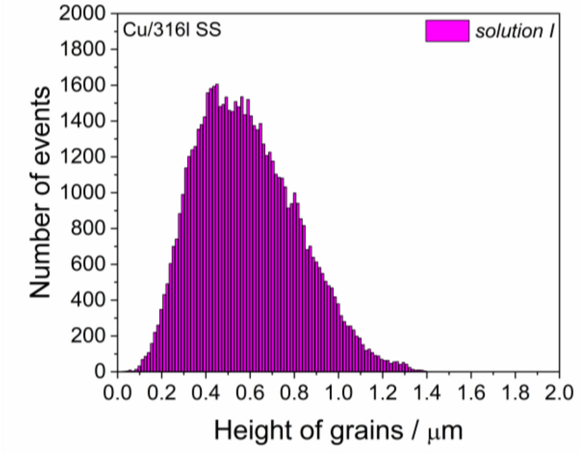

(**a**)

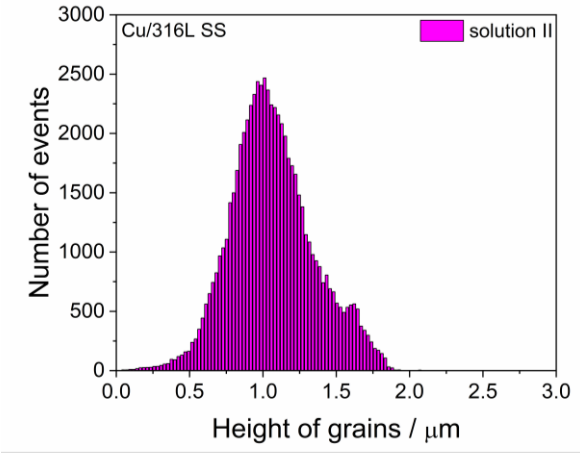

(**b**)

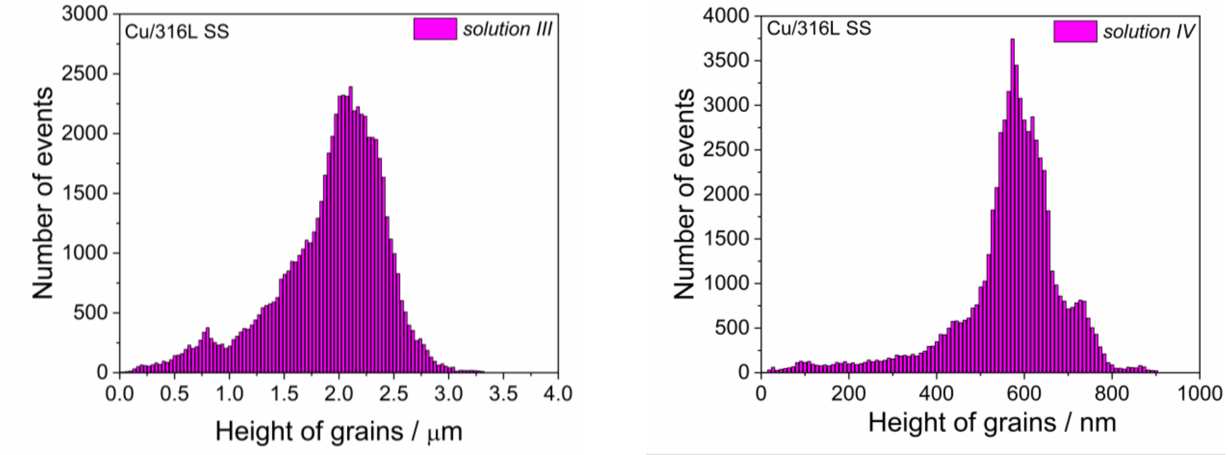

(**c**) (**d**)

Figure S2. The histograms of the height of grains distribution for Cu films obtained by the ED processes on the 316L SS cathode from: (**a**) solution I, (**b**) solution II, (**c**) solution III, and (**d**) solution IV.

S2.5. XRD analysis

Determination of the "Texture Coefficient", *TC*(*hkl*) and the "Relative Texture Coefficient", *RTC*(*hkl*) by analysis of the XRD data.

The ratio of reflection intensity (*hkl*) to the sum of all intensities of the recorded reflections, *R*(*hkl*), (in %) is given by Equation (S1) [64]:

$$R(hkl) = \frac{I(hkl)}{\sum_{i}^{4} I(h_i k_i l_i)} \times 100 \tag{S1}$$

where *I* (*hkl*) is a reflection intensity (*hkl*) plane, in cps, and $\sum_{i}^{4} I(h_i k_i l_i)$ is the sum of all intensities of the recorded reflections, in cps, for the film being considered (in the case of Cu, it is four).

The "Texture Coefficient", *TC*(*hkl*), for every (*hkl*) reflection is defined by Equation (S2):

$$TC(hkl) = \frac{R(hkl)}{R_s(hkl)} \tag{S2}$$

where $R_s(hkl)$ is defined in the same way as given by Equation (S1) but is related to the standard for metal under consideration. This coefficient gives accurate quantitative information about the absolute reflection intensity. The values of *TC*(*hkl*) coefficients larger than 1 indicate the existence of the preferred orientation in given crystal plane.

Finally, the "Relative Texture Coefficient", *RTC*(*hkl*) is defined by Equation (S3):

$$RTC(hkl) = \frac{TC(hkl)}{\sum_{i}^{4} TC(h_i k_i l_i)} \times 100 \tag{S3}$$

The *RTC* (*hkl*) coefficient defines the reflection intensity (*hkl*) relative to the standard (included in the *TC* values).

Since four crystal planes are analyzed, the values of *RTC*(*hkl*) coefficients larger than 25% indicate the existence of the preferred orientation in given crystal plane.

Table S2. Texture calculations for the Cu/Mo films produced galvanostatically from solutions without/with additives (*solution I, II, III, IV*) at a current density of 60 mA cm^{-2}. *R*—Intensity of the diffraction peak; *TC*—Texture Coefficient; *RTC*—Relative Texture Coefficient; s—Cu standard.

Crystal plane	(111)	(200)	(220)	(311)
R_s/ %	54.6	25.1	10.9	9.4
		Cu/Mo/I		
R / %	41.6	21.1	24.7	12.6
TC(*hkl*)	0.76	0.84	2.27	1.34
RTC(*hkl*)/ %	14.6	16.1	43.6	25.7
		Cu/Mo/II		
R / %	46.1	24.1	17.1	12.7
TC(*hkl*)	0.84	0.96	1.57	1.35
RTC(*hkl*)/ %	17.8	20.3	33.3	28.6
		Cu/Mo/III		
R / %	58.0	17.6	14.8	9.60
TC(*hkl*)	1.06	0.70	1.36	1.02
RTC(*hkl*)/ %	25.6	16.9	32.9	24.6
		Cu/Mo/IV		
R / %	25.8	64.1	7.70	2.40
TC(*hkl*)	0.47	2.55	0.71	0.26
RTC(*hkl*)/ %	11.8	63.9	17.8	6.50

Table S3. Texture calculations for the Cu/316L SS films produced galvanostatically from solutions without/with additives (*solution I, II, III, IV*) at a current density of 60 mA cm^{-2}. *R*—Intensity of the diffraction peak; *TC*—Texture Coefficient; *RTC*—Relative Texture Coefficient; s—Cu standard.

Crystal plane	(111)	(200)	(220)	(311)
R_s/ %	54.6	25.1	10.9	9.4
		Cu/316L SS/I		
R / %	21.9	17.9	43.8	16.4
TC(*hkl*)	0.40	0.71	4.02	1.74
RTC(*hkl*)/ %	5.8	10.3	58.5	25.4
		Cu/316L SS /II		
R / %	59.1	19.2	13.3	8.4
TC(*hkl*)	1.08	0.76	1.22	0.89
RTC(*hkl*)/ %	27.3	19.2	30.9	22.6
		Cu/316L SS /III		
R / %	61.6	16.2	12.7	9.50
TC(*hkl*)	1.13	0.65	1.17	1.01
RTC(*hkl*)/ %	28.5	16.4	29.5	25.6
		Cu/316L SS /IV		
R / %	46.0	33.9	13.2	6.90
TC(*hkl*)	0.84	1.35	1.21	0.73
RTC(*hkl*)/ %	20.3	32.7	29.3	17.7

S2.6. Confirmation of poor adhesion via scratch-tape adhesion test

The validation of the bending adhesion test method was done with a standard test method with tape known as "scratch-tape adhesion test”.

For films with expected poor adhesion to the substrate, it is suitable a standard "scratch-tape adhesion test," method B, referred in the literature as the cross-cut tape test [20, 65]. It is based on the ASTM D3359 standard [20, 65]. The basis of this test is as follows: a lattice of 1.0×1.0 mm^2 squares (six cuts per direction) was made on the film, covered with a pressure-sensitive tape (3M), kept for 90 s, and then fast removed from the film. Based on a delamination of the film from the substrate, and its keeping on the tape, the films are classified from 5B (no delamination) to 0B (>65% delamination) [20, 65].

All Cu films analyzed in this study showed a delamination greater than 65%, so they were classified as 0B. It confirmed a weak adhesion of produced films, and therefore, successful use of both Mo and 316L SS as possible substrates in electrolytic production of Cu foils.

Figure S3 shows the Cu film from solution IV on 316L SS cathode before delamination (Figure S3a), and after peeling with 3M tape (Figure S3b), where it is clearly visible that the copper film was delaminated 100%. The compact separate Cu foil is visible on the 3M tape (Figure S3c).

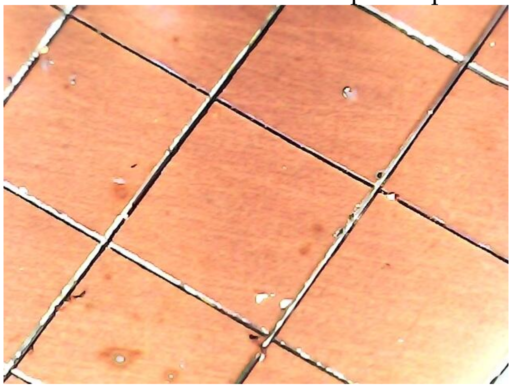

(**a**)

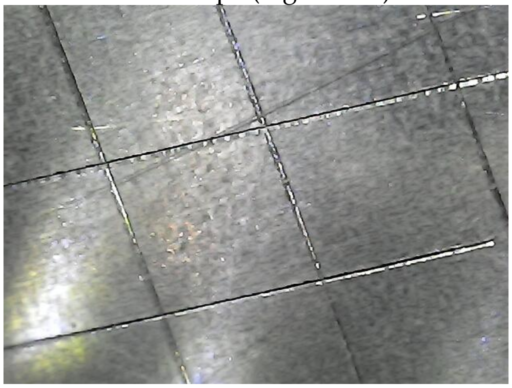

(**b**)

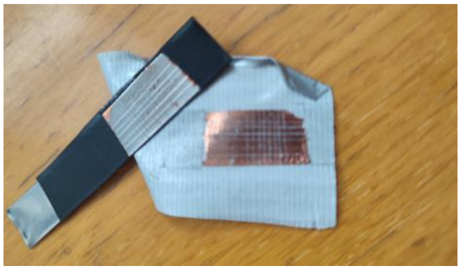

(**c**)

Figure S3. The scratch-tape adhesion test: (**a**) Cu film on 316L SS cathode before delamination, (**b**) 316L SS cathode after pilling Cu film, and (**c**) delaminated Cu foil on the 3M tape.
